# Endotracheal Intubation by Paramedics Using Neodymium Magnet and Modified Stylet in Simulated Difficult Airway: A Prospective, Randomized, Crossover Manikin Study

**DOI:** 10.1155/2019/5804260

**Published:** 2019-10-15

**Authors:** Sedat Bilge, Onur Tezel, Yahya Ayhan Acar, Guclu Aydin, Attila Aydin, Gokhan Ozkan

**Affiliations:** ^1^Department of Emergency Medicine, Gulhane Medicine Faculty, Health Sciences University, Ankara 06010, Turkey; ^2^Department of Emergency Medicine, Gulhane Training and Research Hospital, Health Sciences University, Ankara 06010, Turkey; ^3^Miaclinics, Atasehir, Istanbul 34758, Turkey; ^4^Department of Anesthesiology and Reanimation, Gulhane Training and Research Hospital, Health Sciences University, Ankara 06010, Turkey

## Abstract

**Background:**

The present study evaluates the success and efficacy of endotracheal intubation (ETI) using a modified intubation stylet and a magnet system to direct the stylet into the trachea. The system was developed by the researchers in an attempt to increase the success and efficacy of ETI.

**Methods:**

ETI procedures were performed on an airway management manikin by emergency medical technicians with at least four years of experience in ETI. The technicians used a stylet modified with an iron ball affixed to the tip and a neodymium magnet, designed specifically for the study. The intention was to guide the endotracheal tube into the trachea at the level of the thyroid and cricoid cartilages on the manikin with the aid of the modified stylet and the magnetic force of the neodymium magnet. The success rate, completion time, and degree of difficulty of two procedures were compared: magnetic endotracheal intubation (METI) and classic ETI (CETI).

**Results:**

The success rate was 100% in both groups. The mean completion times for the METI and CETI procedures were 18.31 ± 2.46 s and 20.01 ± 1.95 s, respectively. There were significant differences in completion time and degree of difficulty between the METI and CETI procedures (both *p*=0.001).

**Conclusions:**

We found the use of a neodymium magnet and modified stylet to be an effective method to guide the endotracheal tube into the trachea. The present study may provide a basis for future studies.

## 1. Introduction

Endotracheal intubation (ETI) is the optimum method for protecting the patency of the airway and maintaining oxygenation and ventilation in patients requiring advanced life support [1–3]. Various factors can complicate securing the airway, such as anatomical variations in the airway and related structures and cervical immobilization [4–8].

To overcome the factors complicating ETI procedures, noninvasive intubation methods and devices are available, such as intubating laryngeal mask airways, video-assisted intubation, fiberoptic intubation, and laryngoscope-based lighted stylets [2, 9–11]. The use of invasive methods, such as retrograde ETI and surgical airway management, can lead to increased costs, loss of time, and various catastrophic complications that require management [12, 13]. Although magnetic ETI (METI) techniques have been described in the literature, some limitations have also been reported [14–16]. They are not widely applicable in clinical practice due to these limitations. We aimed to optimize METI use in difficult airway management. An intubation stylet combined with a metal ball was developed by the researchers. A neodymium magnet was used with this modified intubation stylet (MIS). This study aimed to compare the procedural success rate, degree of difficulty, and completion time of this technique with those of classic ETI (CETI).

## 2. Materials and Methods

### 2.1. Study Design

This study was approved by the Gulhane Ethics Board of the University of Health Sciences on May 22^nd^, 2018 (meeting no: 2018/7; decision number: 18/129). Written voluntary informed consent has been obtained from all participants before performing the procedures. The study was designed as a randomized, crossover manikin study.

### 2.2. Participants

All interventions were performed by 20 ambulance and emergency care technicians who were graduates of the Gulhane Military Medical Academy Noncommissioned Officer Health College. All participants had more than four years of emergency medical service experience and were trained in advanced cardiac life support and advanced trauma life support. The operators were given refresher training lasting for at least 30 minutes on the ETI procedure by specialists in emergency medicine prior to the study. These training programs (theoretical and practical) included anatomical landmarks and standard procedural tools on ETI.

### 2.3. Equipment

An airway management manikin (Laerdal Airway Management Trainer; Laerdal Medical, Stavanger, Norway, 2013) was used in the study, fitted with a rigid cervical collar (Ambu® Perfit ACE extrication collar; Ambu Inc., Columbia, MD, USA). The distance between the skin and trachea at the vocal cord level of the airway management manikin was measured as 18.5 mm using the image obtained via fluoroscopy (Infinix-i Core INFX-8000F; Canon Medical Systems, Japan) and the device's computer. The Macintosh (MAC) blade (no. 3) laryngoscope (Teutotechnik Inc., Germany) with a laryngoscope handle and a size 8 cuffed endotracheal tube (ETT) (Kaishou Inc., China) was used in all ETI procedures. Lubricant gel, a bag valve mask (BVM), and a 20 ml syringe were also used to inflate the cuff.

**Figure 1:**
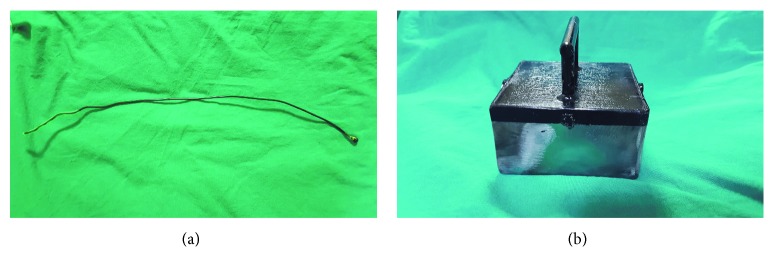
(a) Modified intubation stylet produced by soldering an iron-rich metal ball onto the tip of a standard stylet; (b) the neodymium magnet designed for the study.

The classic intubation stylet (CIS) was a 12 Ch-sized stylet with a length of 40 cm and made of malleable aluminum covered with a synthetic plastic polymer (polyvinyl chloride) (Plasti-med Inc., Turkey). The MIS comprised an iron-rich metal ball measuring 5 mm in diameter soldered to the tip of the classic stylet (Figure 1(a)). The neodymium magnet used was manufactured specifically for this study (Manyet Manyetik Tutucular Inc., Ankara, Turkey). The properties of the magnet reported by the manufacturer were as follows. The magnetic plate was composed of four 40 × 40 × 40 mm grade N52 magnets. The magnets were attached to an ST52 iron plate and covered with 2 mm of stainless protective tin. The grade N52 magnetic plate had a 14,400 Gauss magnetic field, producing a 4,500 Gauss magnetic field on the stainless plate (Figures 1(a) and 1(b)). The holding power applied by the magnet on the MIS was measured by simple dynamometry in the physics teaching laboratory of Middle East Technical University. All devices were used according to the manufacturers' instructions.

### 2.4. Study Protocol

The operators received theoretical and practical training before the study for 60 minutes on three-dimensional laryngeal anatomy, standard laryngoscopy, and the use of the MIS and the N52 neodymium magnet. At the end of the training, the participants were given the opportunity to practice on a model for a total of 10 minutes, with five minutes set aside for each technique. A difficult airway was simulated by fitting a cervical collar to the ETI model. Only one intervention attempt was allowed for each technique.

**Figure 2:**
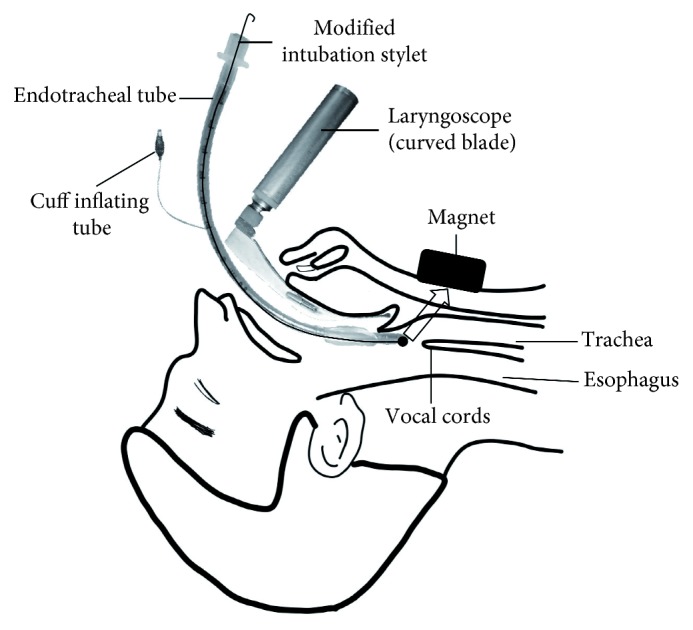
Illustration of endotracheal intubation performed with an endotracheal tube fitted over a modified intubation stylet and a neodymium magnet placed on the anterior aspect of the neck at the level of the thyroid and cricoid cartilages.

- CETI: intubation performed with an ETT fitted with a CIS
- Steps of the CETI procedure: (i) A CIS-fitted ETT was ready on the table with the laryngoscope blade attached before the procedure. (ii) The practitioner started the CETI procedure with the help of the laryngoscope when a “start” command was issued by the researcher. (iii) The CIS was withdrawn by the practitioner, the BVM was attached to the ETT, and the procedure was terminated when lung inflation was detected.
- METI: intubation performed with the MIS, with a neodymium magnet placed on the anterior aspect of the neck at the level of the thyroid and cricoid cartilages (Figure 2)
- Steps of the METI procedure: (i) An MIS-fitted ETT was ready on the table with the laryngoscope blade attached before the procedure. (ii) The practitioner started the METI procedure with the help of the laryngoscope when the researcher issued a “start” command. (iii) The laryngoscope blade was inserted into the mouth, and the laryngoscope was placed by the practitioner while the magnet was simultaneously placed on the anterior aspect of the neck at the level of the thyroid and cricoid cartilages by the operator. (iv) The practitioner pushed the MIS-fitted ETT slowly by holding it gently to direct the magnet's attraction force towards the trachea. (v) The magnet was pulled by the practitioner while removing the laryngoscope from inside the mouth. (vi) The MIS was withdrawn by the practitioner, the BVM was attached to the ETT, and the procedure was terminated when lung inflation was detected. The METI procedure was simultaneously recorded with a video camera under fluoroscopy (Infinix-i Core INFX-8000F; Canon Medical Systems, Japan). A video showing the METI procedure is also available or downloadable at http://bit.ly/2KHFQt6.

**Figure 3:**
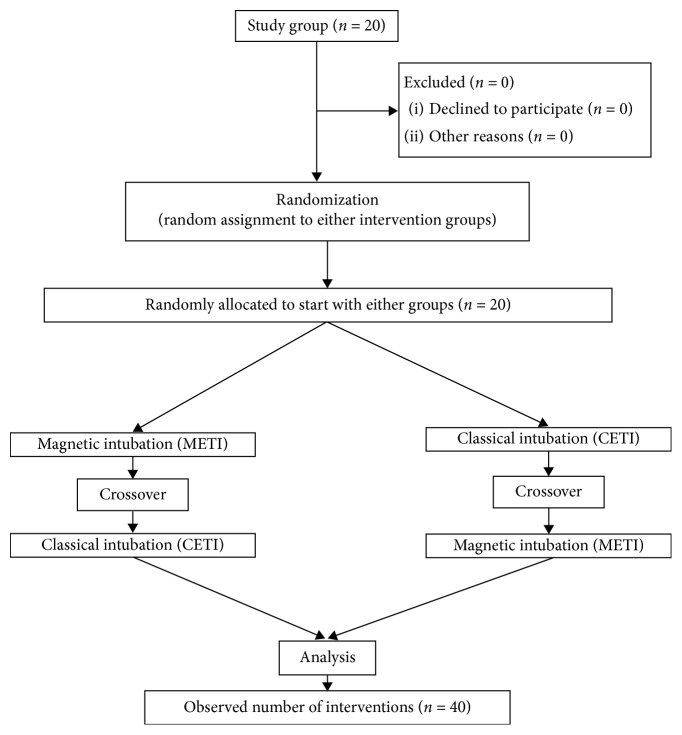
Flowchart of the study.

All participants were randomized to the METI or CETI group prior to the study using an online tool (http://www.randomizer.org). The flowchart of the study is given in Figure 3.

The 30-second limit was determined as the ETI success criterion based on the available literature [17, 18]. For each procedure, the intubation attempt was considered unsuccessful if the procedure could not be performed within 30 seconds. No second attempts were allowed. For interventions using either of the two methods, the criteria for successful intubation were the completion of the procedure by the operator within 30 seconds and the observation of lung inflation on the manikin by the investigators. The completion time was defined as the interval between when the “start” instruction was issued by the investigators to when the BVM was attached to the ETT. The primary endpoint of the study was successful intubation, and the secondary endpoints were completion time and the degree of difficulty reported by the operators. Feedback was received from the operators regarding the degree of difficulty of the procedure, rated using a numeric rating scale (not difficult at all: 0; the most difficult: 10).

### 2.5. Power Analysis

In the power analysis, based on previous study findings (completion time 14.46 ± 2.31 s) [19], assuming a completion time of 17.0 s for ETI with an 80% power and a two-sided error margin of 0.05, 18 participants were required. Thus, we planned to include a total of 20 participants who would be randomized to the study groups.

### 2.6. Statistical Analyses

Statistical analyses were performed using SPSS for Windows version 22.0 (SPSS Inc., Chicago, IL, USA). Descriptive data are expressed as the mean ± standard deviation. A Kolmogorov–Smirnov test was used to assess if the data were normally distributed. The Student's *t*-test was used to compare normally distributed paired groups, and the Wilcoxon rank sum test was used to compare the degree of difficulty between the procedures. A *p* value <0.05 was considered statistically significant.

## 3. Results

The holding power applied by the neodymium magnet to the iron ball fixed to the tip of the MIS was measured from 1 cm to 6 cm using a calibrated dynamometer and recorded as approximately 0.4 N at 6 cm and 2.45 N at 1 cm (Table 1). A total of 20 paramedics (100%) successfully performed 40 interventions, 20 each in the METI and CETI groups; there were no cases of failed intubation. There was a statistically significant difference in completion time between the METI and CETI groups, with the mean completion time being lower in the METI group (*p*=0.001) (Table 2). A comparison of the degree of difficulty rated using the numeric rating scale showed lower scores in the METI group, and the difference between the groups was statistically significant (*p*=0.001) (Table 2).

## 4. Discussion

Alter et al. assessed ETI attempts by paramedics in the prehospital care of 2,299 patients using direct laryngoscopy (DL) with either MAC or miller (MIL) blades and reported that ETI was successfully performed with DL using a MAC blade (*n* = 1865), MIL blade (*n* = 367), and both blades (*n* = 67). The first-pass success rates of ETI performed by paramedics with MAC and MIL blades were reported to be 86% and 73%, respectively [20]. The success rate of CETI in the present study (100%) was higher than that reported by Alter et al. The operators (*n* = 20) in the present study were selected from among paramedics who are responsible for ETIs as part of trauma resuscitation teams in the emergency department of a tertiary training and research hospital, admitting 1,200 emergency patients daily, and who had at least four years of professional experience. The paramedics participate in difficult airway management both in in-hospital and out-of-hospital service. Especially in trauma patients, intubation in the presence of a neck collar is a common procedure they encounter. The high procedural success rate is considered to be related to the experience of the operators in ETI. The high success rate of CETI, similar to that reported in the literature, and the high success rate of METI suggests that METI does not complicate the ETI procedure; therefore, it can be considered as a feasible technique for in-hospital or out-of-hospital settings.

In a study conducted by Maartens et al. involving paramedics as operators, the mean time for intubation was 39.33 s (95% confidence interval, 21.21–57.46) for DL and 50.93 s (95% confidence interval, 17.37–84.50) for VL on a model fitted with a cervical collar [5]. In a study conducted by Kim et al. comparing the MAC laryngoscope and a custom-made ergonomic laryngoscope in an intubation model, the mean time to intubation was 49.7 ± 37.5 s for the MAC laryngoscope [21]. Both studies involved medical students and student paramedics with limited experience in ETI. The lower mean completion times for CETI and METI in the present study are likely related to the ETI experience of the operators gained in the emergency department of a training and research hospital. Furthermore, the significantly shorter completion time for METI procedures than for CETI procedures supports the effectiveness of this technique. The statistically significant difference of 1.7 seconds between the two methods (shorter in the METI procedure) was not clinically relevant for the METI procedure. However, we evaluate it as meaningful data in that METI is a practical technique, considering that it is easy to apply and the completion time has not been extended.

Kim et al. rated the ease-of-use score (not difficult at all: 5, the most difficult: 1) as 3.5 out of 5 points (meaning the degree of difficulty score was 3.0 out of 10 points) among medical students performing ETI on an intubation model using a MAC laryngoscope [21]. The degree of difficulty/ease-of-use score in the METI group in the present study was 2.45 out of 10 points, which was consistent with the findings of Kim et al. That said, significantly lower ease-of-use scores were recorded for the METI procedure than for the CETI procedure, suggesting that the method described in the present study can be considered useful in the management of difficult airways.

Ezri et al. measured the distance between the skin and trachea at three measurement points using ultrasound and reported mean distances of 17.5 ± 1.8 mm at the level of the vocal cords (measurement point 1) in patients with easy laryngoscopy and 28 ± 2.7 mm in obese patients with difficult laryngoscopy [22]. They also reported mean distances of 22.8 ± 5 mm at the isthmus of the thyroid gland (measurement point 2) in patients with easy laryngoscopy and 25 ± 1.3 mm in obese patients with difficult laryngoscopy. The mean distance at the level of the suprasternal notch (measurement point 3) was 27.4 ± 6.6 mm in patients with easy laryngoscopy and 33.0 ± 4.3 mm in obese patients with difficult laryngoscopy. Prasad et al. evaluated airway structures and their relationship with each other using computed tomography scans and ultrasonography and reported mean distances of 5.48 ± 1.03 cm and 4.15 ± 0.5 cm from the upper border of the hyoid bone to the epiglottis, respectively [23]. The distance between the skin and trachea at the level of the vocal cord was measured as 1.85 cm on the airway management manikin used in the present study. Thus, any diameter from the skin to the trachea can be expected to be less than 6 cm. Therefore, the magnet was aimed to provide effective holding power at up to 6 cm to direct the MIS into the trachea, and it provides sufficient holding power in patients with both easy and difficult laryngoscopies.

Magnetic intubation methods have been reported previously [14–16]. Spencer et al. reported on magnetic orotracheal intubation [14]. In the editorial letter published by Spencer, there was mention of a patented technique (United States Patent no. 4.063.561) using an electromagnet [24]. In this technique, numerous tiny magnetic particles that fit inside the plastic section were placed at the end of the ETT. We could not find any study demonstrating the use of this technique in the current literature. In our prestudy trials, we tested the technique also by placing a magnet on the stylet tip to achieve a stronger attraction force. However, in cases where the magnetic fields of the two magnets overlapped, we observed that with the effect of this magnetic field, the stylet did not move linearly in the desired direction and could deviate from the linear progression axis, rendering the procedure more difficult.

Patil et al. designed a further study and reported the success of that technique [15]. Their model was applied by experienced anesthesiologists and used a 2 × 2 × 1 cm cobalt magnet with an iron stylet. Patil et al. directed the two-part translucent 18-Fr intubation catheter with an iron stylet into the glottis with the help of a 2 × 2 × 1 cm cobalt magnet placed over the trachea. The stylet was withdrawn through the catheter directed to the glottis with the attraction force of the magnet, and an ETT (not less than 7 mm in diameter) was inserted into the trachea over this catheter using the Seldinger technique in nondifficult airway cases under general anesthesia in the operating room. They stated that in order to use this technique successfully in patients under general anesthesia, it is necessary to release the tube in a way that allows the magnet to attract the stylet. The MIS used in our study does not require the use of an additional catheter or tube for ETI nor does it require a second or another maneuver. In our study, an ETT with MIS, instead of a routinely used CIS, was found to enable paramedics to perform the procedure in a single step in in-hospital and out-of-hospital settings. In addition, the MIS, which we designed for our study, is suitable for use with all ETT sizes between 5.5 and 9 and uses a neodymium magnet with a stronger magnetic attraction force instead of a cobalt magnet.

Gaspari et al. studied magnetically guided ETI in an airway dummy and reported that medical students without prior ETI experience successfully applied the technique [16]. Gaspari et al. carried out a magnetic intubation study on a manikin in difficult airway conditions with inexperienced medical students who had no previous ETI experience on human beings. They highlighted the need to perform a trial with experienced practitioners as a limitation of their study. In order to create a force of attraction, a rare earth magnet was attached to the end of a flexible stylet with the attraction force at the level of the cricoid cartilage of the manikin. When two magnets are normally placed on a flat surface, the opposite poles immediately attract each other and the magnetic fields of the like poles of two magnets repel each other. However, if a magnet is attached to the end of the stylet in the ETT or a magnetic structure is placed at the end of the ETT, the ETT does not move directly across towards the magnet on the anterior face of the neck as it would on a flat surface. Since the pathway of the ETT is anatomically curved, the forward push of the ETT may cause the repulsion/deflection of the same poles of the opposing magnets in the oropharyngeal and supraglottic regions until the opposite poles of the opposing magnets meet each other and generate a force of attraction. Furthermore, the magnetic type stylet can get attached to the metal blade of the laryngoscope when the ETT is pushed forward in the oropharyngeal region. In this case, deviations may occur in the linear progression of the ETT. To prevent these deviations and to ensure the linear progression of the ETT, in our study, we combined the N52 neodymium magnet with a metal ball-tipped aluminum stylet to increase the attachment force and direct the ETT towards the trachea. In this respect, the MIS and METI procedure designed for our study differs from the magnetic type stylet used by Gaspari et al. Moreover, in their study, Gaspari et al. did not clearly address the issue concerning the suitability of the internal diameters of the magnetic type and the ETT to be used.

Patil et al. and Gaspari et al. did not include any control groups in their studies, which was a major limitation. In our study, we compared the METI group with the CETI as a control group and determined which paramedics could successfully apply magnetic intubation. In addition, the combination of the N52 neodymium magnet and a metal ball-tipped aluminum stylet increased the holding power and directed the ETT towards the trachea.

Our primary target, when designing the spherical stylet tip was to avoid damaging the soft tissues during METI. The formation of the iron-rich metal ball, with a larger surface area than the diameter of the stylet, increased the holding power of the magnet. We aimed to hold the MIS from a distance of at least 6 cm with a 4 × 4 × 4 cm sized N52 neodymium magnet. However, this could not be achieved with either a magnet-tipped stylet or a ferrite magnet, based on our prestudy experience. In addition, during the prestudy trial, we observed that with the use of magnet-tipped stylets, the tip of the ETT deviated from the linear direction due to the dual magnetic fields before reaching the vocal cords. We suggest that an iron-rich metal ball should be attached to the tip of the stylet to provide this linear movement.

## 5. Limitations

The major limitation of the present study was that the ETI procedures were performed on an intubation model and that other conditions that could complicate the management of the airway, such as obesity and gastric content, were not simulated. Thus, this approach did not provide sufficient data for clinical use.

## 6. Conclusions

METI has the potential for use as an alternative method for the management of difficult airways. It is more advantageous than CETI in terms of procedure completion time. Furthermore, the lower difficulty score of METI compared to CETI has the potential to minimize hesitation in deciding to perform and in performing an ETI. The METI method could be further improved through the use of smaller but stronger specially manufactured magnets and with future studies on cadavers and human subjects.

## Figures and Tables

**Table 1:** The holding power of the magnet on the intubation stylet measured from various distances.

Distance (cm)	Measurement 1 (N)	Measurement 2 (N)	Mean (N)
6	0.40	0.40	0.40
5	0.55	0d.60	0.58
4	0.80	0.80	0.80
3	1.20	1.20	1.20
2	1.70	1.65	1.68
1.5	2.10	2.10	2.10
1	2.40	2.50	2.45

cm, centimeter; N, newton.

**Table 2:** Comparison of the procedure completion times and degree of difficulty between the two groups.

Completion time (seconds)				Numeric rating scale^#^	
Mean ± SD		*p* ^*∗*^	95% CI	Mean ± SD	*p* ^*∗∗*^
METI	18.31 ± 2.46	0.001	0.94–2.46	2.45 ± 0.76	0.001
CETI	20.01 ± 1.95			4.20 ± 0.83	

^*∗*^Student's *t*-test; ^*∗∗*^Wilcoxon rank sum test.

## Data Availability

The data that support the findings of this study are available from the corresponding author upon reasonable request.
